# Natural products in drug discovery and development: Synthesis and medicinal perspective of leonurine

**DOI:** 10.3389/fchem.2022.1036329

**Published:** 2022-10-17

**Authors:** Zhaoyi Li, Keyuan Chen, Peter Rose, Yi Zhun Zhu

**Affiliations:** ^1^ State Key Laboratory of Quality Research in Chinese Medicine, School of Pharmacy, Macau University of Science and Technology, Taipa, Macau, China; ^2^ School of Biosciences, University of Nottingham, Nottingham, United Kingdom; ^3^ Shanghai Key Laboratory of Bioactive Small Molecules, Department of Pharmacology, School of Pharmacy, Fudan University, Shanghai, China

**Keywords:** herb leonuri, leonurine, synthesis, pharmacological effects, cardiovascular diseases, nervous system diseases

## Abstract

Natural products, those molecules derived from nature, have been used by humans for thousands of years to treat ailments and diseases. More recently, these compounds have inspired chemists to use natural products as structural templates in the development of new drug molecules. One such compound is leonurine, a molecule isolated and characterized in the tissues of *Herb leonuri*. This molecule has received attention from scientists in recent years due to its potent anti-oxidant, anti-apoptotic, and anti-inflammatory properties. More recently researchers have shown leonurine to be useful in the treatment of cardiovascular and nervous system diseases. Like other natural products such as paclitaxel and artemisinin, the historical development of leonurine as a therapeutic is very interesting. Therefore, this review provided an overview of natural product discovery, through to the development of a potential new drug. Content will summarize known plant sources, the pathway used in the synthesis of leonurine, and descriptions of leonurine’s pharmacological properties in mammalian systems.

## Introduction

Human civilization, across all continents, has a long history of use of natural products either in the form of plant, fungal, microbial, or animal-derived extracts, preparations, or isolated compounds. These preparations are being used in the treatment of various ailments and diseases (Ji et al., 2009). Examples of developments in this field litter the historical records in various research publications and pharmacopeias. Common examples include the18th-century description by Europeans of the discovery of aspirin in the leaves of the willow tree (*genus Salix*), having properties that reduce pain, fever, and inflammation (Ugurlucan et al., 2012). Similarly, Paclitaxel, a popular anticancer drug, that was first isolated from the bark and needles of Taxus brevifolia in 1971, and now approved by the FDA for the treatment of various types of cancer (Zhu and Chen, 2019). Even today, natural products derived from various plants species are still a valuable source of lead compounds, and this is inspiring a generation of scientists interested in the development and design of new therapeutic drugs (Katz and Baltz, 2016). Many of these compounds have various biological activities (Dutta et al., 2019) including anti-inflammation (Azab et al., 2016), anti-cancer (Liu et al., 2020), anti-oxidation (Jaganjac et al., 2021), and anti-viral properties (Thomas et al., 2021). Here, we draw on some of these examples, and describe various success stories relating to the development of natural products as drugs. This review will cover paclitaxel, artemisinin, aspirin, and camptothecin, and we summarize the unique aspects of their developmental process. In addition, a description will be given relating to plant sources, synthetic pathways, and pharmacological activities of the natural product, leonurine. Leonurine has gained interest from scientist due to its therapeutic potential in the treatment of cardiovascular and neurological diseases.

## Extraction and separation

Plants, fungi, microorganisms, and some animal species are novel sources of natural products, and tissues form these have been exploited by researchers in their search for new therapeutics (Sen and Samanta, 2014; Beutler, 2019). Due to the diverse chemical structures and differences in stability and physicochemical properties of natural products, extraction and separation methodologies of natural products have always been a huge challenge. Indeed, difficulties in extraction procedures are a common topic of discussion in the early phases of research on natural products (Sarker and Nahar, 2012; Wang et al., 2022). This problem has led to the development of numerous forms of extraction and isolation procedures used by natural product chemists, ranging from basic solvent extraction procedures through to supercritical CO_2_ extraction methods; each has its challenges. Historically, solvent extract procedures have been described since the 17th century. Scientists used solvent extraction techniques to isolated morphine from the milk of poppies (Brook et al., 2017), quinine from the bark of the Cinchona tree, and cocaine from coca leaves (Goldstein et al., 2009; Achan et al., 2011). In addition to extraction methods, further complexity arises when the compound of interest requires separation from other constituents present in the tissues of the natural source. For this reason, separation methods such as column chromatography have been developed, and these are often coupled to some form of screening technique to ensure the molecules of interest are present in separated fractions. For example, a biological assay or compound confirmation assessment like nuclear magnetic resonance (NMR) or mass spectroscopy (Chun-Sheng et al., 2016). Common separation methods include high performance liquid chromatography (HPLC) or more informed approaches like liquid chromatograph mass spectroscopy (LC-MS) that can be utilized to establish a picture of compound composition. For example, HPLC has been used to conduct fingerprint analysis of compounds having free radical scavenging activities in Angelica sinensis (Yang, 2013).

**FIGURE 1:**
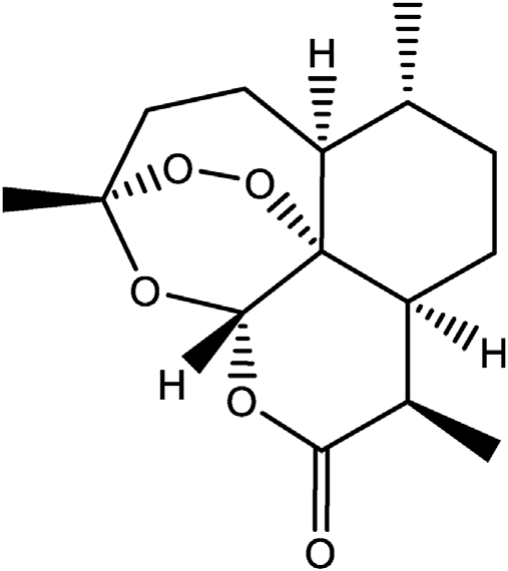
The chemical structures of Artemisinin.

One of the success stories in natural product chemistry is the extraction and isolation of the antimalarial drug, artemisinin. Artemisinin is an example of a sesquiterpene lactone and was first extracted from the plant sweet wormwood (Figure 1) (A.s.r group, 1977). Isolation and characterization were conducted in the laboratory of Tu Youyou, who won the Lasker Prize in Clinical Medicine in 2011, and later the Nobel Prize for Medicine in 2015. Initial work found that extracts of *Artemisia annua* obtained by heating of plant tissues had minimal antimalarial effects. Therefore, researchers began to interrogate the earliest historical reference to *Artemisia annua* in Ge Hong’s “Elbow Reserve Emergency Recipe”. These records revealed a more efficient extraction method *viz. A. annua* immersed in water to obtain a juice. This method avoided heating, and yielded extracts with effective anti-malarial properties. Researchers then modified the extraction process in view of the historical information and later used low temperature extraction procedures to isolated the, active ingredients (Tu, 2016; Wang et al., 2019). Finally, the extracts were separated to obtain artemisinin and analogues allowing for further structural confirmation (White et al., 2015; Chang, 2016; Xia et al., 2020). In addition to antimalarial activity, artemisinin also has antiviral (Liu et al., 2019), antitumor (Slezáková and Ruda-Kucerova, 2017), anti-inflammatory (Zhang et al., 2021), and other pharmacological activities, and has a certain therapeutic effect on autoimmune diseases (Efferth and Oesch, 2021). This example of the isolation and characterization of artemisinin draws on the appreciation of historical and traditional knowledge. And allowed for the optimization of methods to facilitate the extraction of artemisinin now widely used in the treatment of malaria.

Advances in analytical chemistry and organic synthetic routes, extraction and separation techniques have allowed for new approaches to be used by natural product chemistry that give higher extraction efficiency and greater yields. For example, carnosic acid and rosmarinic acid from rosemary were obtained by supercritical fluid extraction (Lefebvre et al., 2021), and brassin from *Caesalpinia sappan* was separated by high-speed countercurrent chromatography (He et al., 2020).

## Artificial synthesis and structural modification

On occasion, researchers are faced with the problem that they are unable to obtain plant tissues in large quantities for the extraction of molecules of interest or that traditional extraction approaches are not efficient enough to obtain compounds at usable levels. Therefore, by the middle of the 19th century, synthetic organic chemistry provided an alternative route to obtaining natural products, albeit *via* a synthetic chemical means. This is exemplified by the breakthrough in production of the first synthetic drug, chloral hydrate (Jones, 2011). This breakthrough spawned a new era in drug development and paved the way for the production of other biological active molecules with the capacity to produce molecules on an industrial scale (Crane and Gademann, 2016).

**FIGURE 2:**
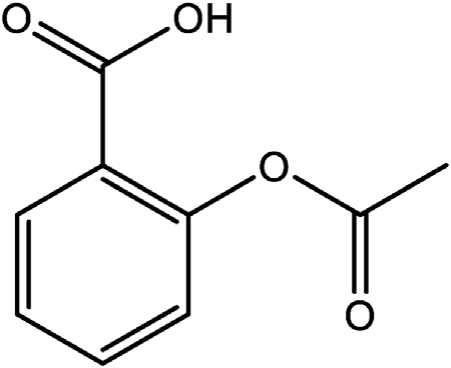
The chemical structures of Aspirin.

Aspirin, also known as acetylsalicylic acid, exerts an anti-inflammatory effect by inhibiting the production of prostaglandins and thromboxanes in mammalian cells and tissues, and is widely prescribed as an anti-inflammatory, pain relief and fever reducing medication (Vane, 1971; Montinari et al., 2019). It is one of the most widely used chemically synthesized drugs in the world (Montinari et al., 2019). Interestingly, the origin of aspirin can be traced back 3,500 years ago to the use of willow bark as an ancient pain reliever and antipyretic drug. The active ingredient salicin is now chemically synthesized (Mann, 2000; Montinari et al., 2019). Salicin and other natural derivatives offer examples of how synthetic approaches can be used to im-prove on the original molecule. In many instances, therapeutics are developed through different structural modifications to reduce toxicity, or to improve the physicochemical traits of compounds such as poor water solubility; this affording better candidate drugs (Yao et al., 2017; Solís-Cruz et al., 2021). Chemically synthesized salicylates are known to cause nausea, stomach irritation and ringing in the ears as side effects. Therefore, to solve this problem, sodium salicylate was modified using acetyl chloride to synthesize acetylsalicylic acid, more commonly known commercially as aspirin (Figure 2) (Montinari et al., 2019; Valgimigli, 2019). This simple modification reduced some of the side-effects attributed to this compound. Aspirin was patented in the United States in 1900, and it was successfully marketed 4 years later. The use of synthetic routes of production show that this approach can have advantages over traditional methods of extraction from plant tissues. To date, asprin is the best-selling drug in the world (Montinari et al., 2019; Valgimigli, 2019), that is mainly used as an anti-platelet drug to prevent cardiovascular and cerebrovascular diseases, such as myocardial infarction, thrombosis, and cerebral apoplexy (Desborough and Keeling, 2017).

**FIGURE 3:**
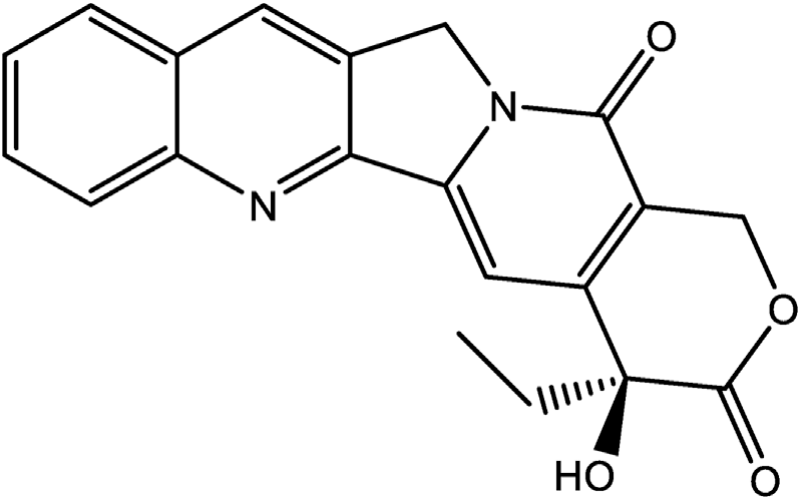
The chemical structures of Camptothecin.

Other natural products are also worthy of mention. The quinoline alkaloid, camptothecin is highly cytotoxic and was first isolated from the bark and branches of *Camptotheca acuminata* in China (Figure 3). Camptothecin has significant antitumor properties and was approved in 1970 for the treatment of gastric cancer, bladder cancer and some leukemias (Wall et al., 1966; Chen and Liu, 1994; Khaiwa et al., 2021).

While camptothecin has a wide range of applications, it is limited due to its poor water solubility, fast hydrolysis rate, high toxicity and issues relating to drug resistance (Li et al., 2006; Li et al., 2017). In order to improve the anticancer efficacy and safety of camptothecin, a series of analogs were synthesized using structural modification (Venditto and Simanek, 2010; Khaiwa et al., 2021). On the basis of retaining the key active structure of camptothecin, chemical modifications focused on changes to key functional groups (Martino et al., 2017). For example, the quinoline ring in the original structure can be opened to convert the molecule into a corresponding ring-opened sodium salt, so as to improve the solubility, and to aid its use in intravenous administration (Ulukan and Swaan, 2002; Martino et al., 2017). Moreover, if the modifications are made on the quinoline ring, the anti-cancer activity can be retained without increasing the cytotoxicity of the molecule. In parallel, by increasing the number of carbon chains in the quinoline ring it is possible to increases lipid solubility and stability in plasma (Liu et al., 2015). Lastly, the hydrolysis of the lactone ring *in vivo* reduces the anticancer activity of camptothecin, by reducing intramolecular hydrogen bond prevents hydrolysis from occurring (Martino et al., 2017).

To date, camptothecin has been used as a structural template for the synthesis of other derivatives namely topotecan, irinotecan and homocamptothecin respectively. Both topotecan and irinotecan have FDA-approval and are both water-soluble derivatives used in the treatment of some clinical cancers (Winterfeldt et al., 1975; Thomas et al., 2004). Topotecan (7-ethyl-10-[4-(1-piperidino)-1-piperidino] carbonyloxycamptothecin) contains a basic amine side chain, which makes it easy to form an ammonium salt and improves water solubility. Topotecan is widely used clinically to treat ovarian cancer and small lung cancer (Liu et al., 2015; Martino et al., 2017). Similarly, irinotecan (9-[(dimethylamino)methyl]-10-hydroxy-camptothecin) is a carbamate analogue of camptothecin, and has enhanced water solubility that is attributed to the presence of an alkaline side chain. Interestingly, irinotecan can be hydrolyzed into metabolites with strong anti-tumor activity *in vivo*, and this drug is currently used in the treatment of rectal cancer (Martino et al., 2017). Collectively, camptothecin is a good example of how structural modification of natural products can be used to manipulate the physicochemical properties of a molecule. On the basis of retaining the original active skeleton, through structural modification, better solubility, greater stability and enhanced anticancer activity can be achieved.

## Drug delivery

As discussed, structural modification of natural products can improve solubility, chemical stability, resistance to metabolism, and to enhance the ability of a drug to cross the blood-brain barrier (Chen et al., 2015; Yao et al., 2017). Occasionally, structural modification of target drug molecules fails to alter bioavailability or to reduce drug toxicity. In this scenario, other strategies are needed to improve and manage the delivery of drugs to cells and tissues. In the last decade, novel drug delivery systems have become popular and include various nano-carriers (Erdoğar et al., 2018; Patra et al., 2018), lipid agents (Efendy Goon et al., 2019), and transdermal delivery systems (Patil and Saraogi, 2014). At present, nanocarriers are one of the most robust delivery systems used in drug research to deliver encapsulate drugs (Wong et al., 2020; Solís-Cruz et al., 2021).

**FIGURE 4:**
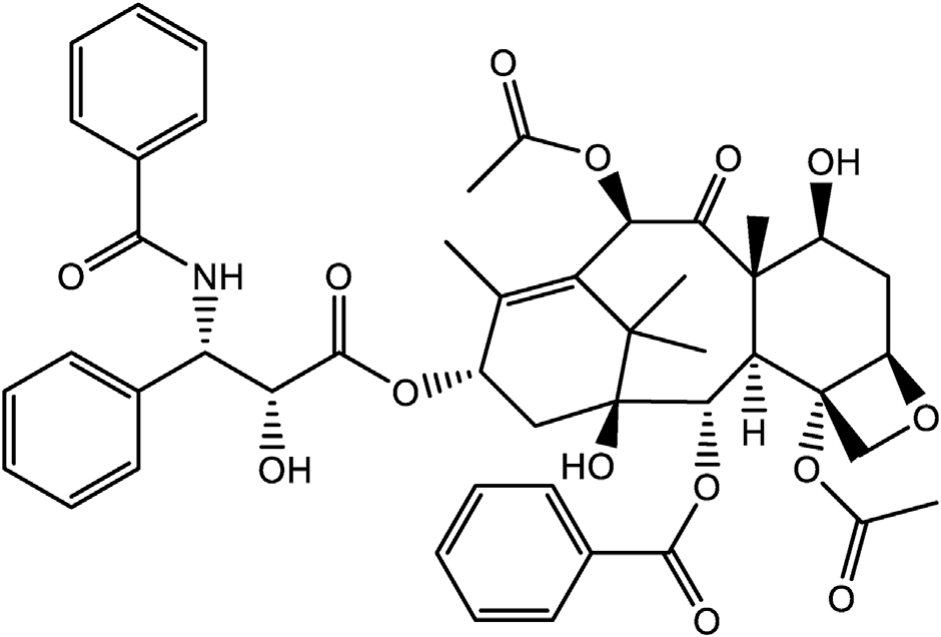
The chemical structures of Paclitaxel.

The natural product, paclitaxel, is a secondary metabolite produced by the *genus Taxus*, and was first isolated from the Pacific yew in 1971 (Figure 4). Due to its strong anticancer activity, it was approved for use by the FDA in 1993 for the treatment of various cancers, such as breast cancer, Ovarian, and lung cancer (Wani et al., 1971; Cragg, 1998; Gallego-Jara et al., 2020). Unfortunately, members of the *genus Taxus* are slow growing species, with plants often taking 200 years to reach an appreciable size *viz.* 40 feet in height. At this size, following harvest, only 0.5 g of paclitaxel could be feasibly extracted from plant tissues. To place this into some context, to treat a single patient requires 2 g of paclitaxel, the equivalent of four mature yew trees. As a result, the supply of paclitaxel was greatly restricted in the early years of its clinical use (Alqahtani et al., 2019; Gallego-Jara et al., 2020). However, as patient demand for paclitaxel grew, new developments were needed to meet the growing demand for this drug. This droves research to identify alternative paclitaxel production methods, including total syn-thetic routes, semi-synthesis, and microbial engineering (Gallego-Jara et al., 2020). Currently, the most commonly used methods of production are semi-synthetic methods (Kumar et al., 2019).

While paclitaxel has strong anticancer activity, its use is made challenging due to its hydrophobic properties and low solubility in water (Bernabeu et al., 2017; Gallego-Jara et al., 2020). To solve this problem, polyoxyethylene castor oil, Cremphor EL (CrEL), and ethanol delivery systems are being developed as novel drug carrier systems. Other approaches are also been considered to facilitate parenteral administration and to reduce adverse reactions like severe allergic reactions (Gelderblom et al., 2001). Currently, albumin-bound paclitaxel (nab-paclitaxel) a nano-delivery approaches have been developed. Nab-paclitaxel, is a formulation that utilises nanoparticles as a carrier without the need for CrEL. Nano-particles are ap-proximately 130 nm in size, and allow for intravenous infusion (Petrelli et al., 2010). Compared with traditional paclitaxel, nab-paclitaxel has reduced side-effects that is largely attributed to the lack of CrEL. This means that higher doses of paclitaxel can be delivered with shorter infusion duration (Gradishar, 2006). In 2005, nab-paclitaxel was approved by FDA for the treatment of metastatic breast cancer (Kundranda and Niu, 2015).

Paclitaxel is a natural product with potent anti-tumor activity. In recent years, a variety of methods have been developed to replace traditional extraction protocols, that circumvent the demand for large batches of raw materials. In addition, by adopting nanotechnology delivery approaches, problems of poor water solubility, low bioavailability, and toxicity have been resolved. These advances have become important in the development of clinical first-line treatment of some cancers by delivering anti-cancer drugs in a more refined manner.

## *Herb leonuri* and the identification and characterization of leonurine

**FIGURE 5:**
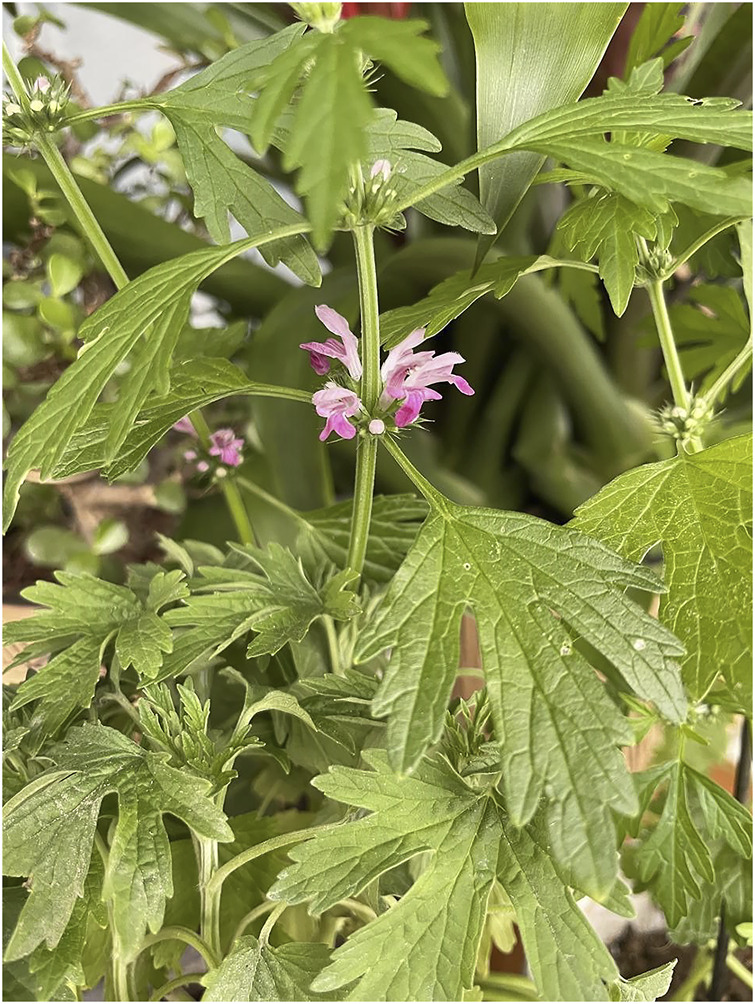
The plant diagram of *Herb Leonuri*.

Stories relating to the development of other natural products are equally as fascinating as that of asprin, camptothecin, and paclitaxel. *Herb leonuri*, commonly known as “Yi-Mu-Cao”, is an annual or biennial herb of the lamiaceae family. The plant is native to parts of China, Central Europe, Scandinavia, and Russia, is now naturalized in Japan, Java, Malaysia, and North America (Zhu et al., 2018). According to the Flora of China, *H. leonuri* has a squarish stem, which is clad in short trichome hairs, and is often purplish in coloration especially near the nodes. The opposite leaves have serrated margins and are palmately lobed with long petioles, basal leaves are wedge-shaped with three points while the upper left have three to five. They are slightly hairs above and greyish beneath, and flowers appear in leaf axils on the upper part of the plant and have three-lobed bracts. The calyx of each flower is bell-shaped and has five lobes, and the corolla is irregular and eight to 12 mm in length. The flowers are pink to lavender, usually with a hairy lower lip. There are four protruding stamens, two short and two long, with one pistil, and the fruit has four-chambers (Figure 5) (Wojtyniak et al., 2013).

**FIGURE 6:**
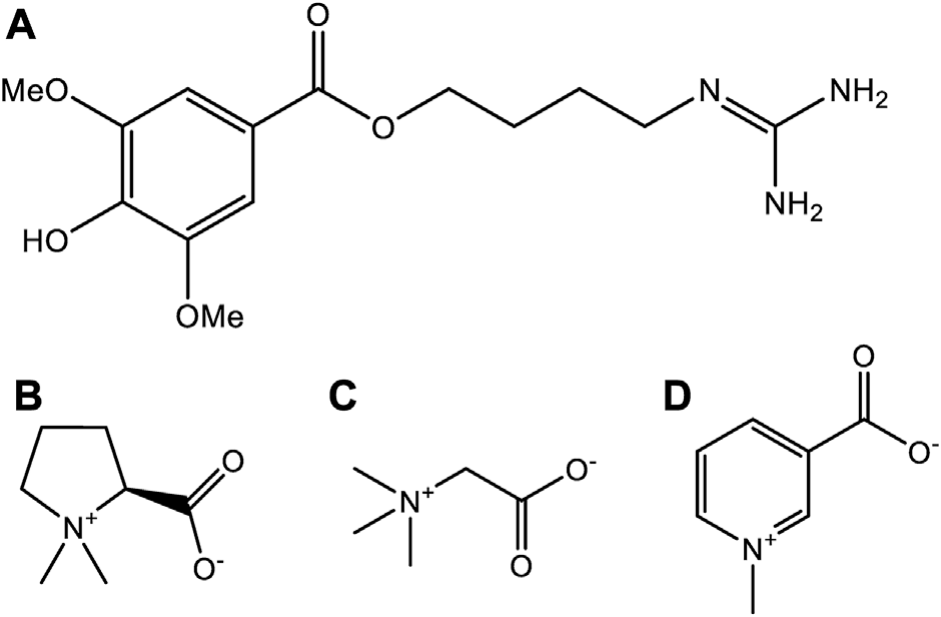
The chemical structures of four alkaloids from *Herb Leonuri*. **(A)** The chemical structures of leonurine. **(B)** The chemical structures of stachydrine. **(C)** The chemical structures of betaine. **(D)** The chemical structures of trigonelline.

According to the record of “Shen Nong’s Materia Medica”, *H. Leonuri* has a pungent taste, and is bitter. The plant is widely used to promote blood circulation, to manage and regulate menstruation, to aid hydration, reducing swelling, clearing heat, and to aid detoxifying (Miao et al., 2019). *H. Leonuri*, as its name is” a beneficial herb for mothers” (Miao et al., 2019), and is considered as a traditional herbal medicine. Other use for this plant includes the treatment of gynecological diseases, irregular menstruation, dysmenorrhea, lochia, edema, oliguria, and Sores (Li et al., 2020). Since 1990, it also has been listed in the Pharmacopoeia of the People’s Republic of China, in which many kinds of traditional Chinese medicine prescription contain this plant species. To date, several active compounds have been identified in tissues and extracts of *H. leonuri* including various alkaloids, flavonoids, diterpenes, iridoid glycosides, sterols, peptides, phenylpropanoids, and phenolic glycosides (Li et al., 2020). Alkaloids are the most important class of active ingredients in this plant, and have become the focus of much research (Zhang et al., 2018). Indeed, four alkaloids have been isolated and characterized in the tissues of this plant namely, leonurine, stachydrine, betaine, and trigonelline, respectively (Figure 6). The pharmacological effects of *H. leonuri* are mainly attributed to the presence of leonurine (Fiskum et al., 2004; Liu X. et al., 2010; Li et al., 2020), a compound present in levels equivalent to 0.02%–0.12% fresh weight (Liu et al., 2013; Huang et al., 2021).

### Isolation and purification aspects of leonurine

Leonurine was first isolated from the plant *H. Leonuri* in 1930, and other alkaloid compounds such as stachydine, betaine, and trigonelline were also isolated. There are various approaches for extracting, isolating, and purifying leonurine from the plant. As early as 1977, Yeung et al. ground and impregnated 5 L of acid methanol (0.1%, v/v) per kilogram of dried plants to obtain a methanol extract of leonurine, and then used alumina column and dextran G-25 column, eluted with a gradient of methanol to 2% acetic acid-methanol, and finally isolated 50 mg/kg of leonurine (Yeung et al., 1977). In 2004, Chao et al. reported the use of ethyl acetate in a Soxhlet extractor to decolorize H. Leonuri and ethanol ultrasonic extraction. The total alkaloids in 2 g crude drug powder accounted for 0.3%, of which stachydine accounted for 0.1–0.2% and accounted for 0.01–0.05% (Zhi et al., 2004). In 2010, Chen et al. reported a method with a high recovery rate, Chen et al. extracted 350 ml of 95% ethanol per 100 g of dry plants for 2 h each, repeated 3 times, and finally obtained 0.15 mg/g of leonurine (Chen et al., 2010). In 2012, Kuchta et al. published a high-performance liquid chromatography method. Kuchta et al. used 120 ml of boiling water to extract 6 g of the plant powder under reflux for 1 h and fixed it with a special octadecyl-bonded stationary phase and an acetonitrile/water gradient as fluidity yielded approximately 3 mg of leonurine (Kuchta et al., 2012). In 2017, Jiang et al. used a two-phase system of ethyl acetate-n-butanol-water (3:2:5) as high-speed countercurrent chromatography to obtain 68 mg of leonurine from 2.48 g of the plant crude extract, and the purity is about 96.2% (Jiang, 2017). In the same year, Cao et al. successfully developed an acidic ionic liquid ultrasonic-assisted extraction method. Cao et al. mixed 1 g of dried plants powder with 20 ml of a 1 mol/L [HMIM][HSO4] aqueous solution, ultrasonicated, and filtered, which could be extracted 0.136‰ of leonurine from plants within 30 min (Cao et al., 2018). This method not only greatly shortens the extraction time, but also reduces the use of organic reagents.

### Structural elucidation and analysis of leonurine

In the past, the traditional identification method of leonurine was to use reverse silica gel thin layer chromatography plate (60 F254) and MeOH:CH_2_Cl_2_:NH_3_ 25% (8:2:3) as mobile phase, under 154 nm UV lamp, leonurine was identified with the Rf value of 0.31 (Kuchta et al., 2012). Nowadays, the identification of leonurine relies more on HPLC, MS, and NMR. Chen et al. used a Acquity UPLC BEH C18 reversed-phase column (100 mm × 2.1 mm) with 1.7 μm spherical porous particles and methanol-ammonium formate (pH = 4.0) as the mobile phase at a flow rate of 0.2 ml/min separation, the maximum absorption peak area of leonurine was detected at 277 nm. Further, under the conditions of the ESI model and typical background source pressure read by ion meter of 1.2 × 10–5 Torr, the capillary temperature of 250 °C, electrospray needle voltage of 4 kV, and drying gas of nitrogen, finally, leonurine was obtained with m/z of 321 and ion fragment m/z of 259, 181, and 114 (Chen et al., 2010). At the same time, Xie et al. also used an Agilent Edlipse Plus C18 (100 mm × 2.1 mm, 3.5 μm) reversed-phase column and methanol–0.1% formic acid solution (20:80, 0.2 ml/min) as the mobile phase. In positive electrospray ionization interface and multiple reaction monitoring modes, m/z 312.2→181.1 was determined to be leonurine (Xie et al., 2015). Li et al. used diphenhydramine as the internal standard on an Agilent ZORBAX Eclipse XDB-C18 column (150 mm × 4.6 mm, 5 μm) and a methanol-water mixture containing 0.1% formic acid as the mobile phase with 0.6 ml/min of flow rate was obtained at the retention time of 6.43 min. Furthermore, leonurine was also determined by m/z of 312.2→181.1 under the reaction monitoring (MRM) mode of multiple transitions for mass spectrometry analysis, which used an Agilent 1,200 series HPLC system and an Agilent 6,410 triple quadrupole mass spectrometer equipped with an electrospray ionization (ESI) source. This method detected leonurine and stachydrine in rat plasma, their lower limits of quantitation were 0.895 ng/ml and 0.287 ng/ml, respectively. The linear relationship coefficient with the calibration curve containing the internal standard exceeded 0.99 (Li et al., 2013).

### Artificial synthesis of leonurine

In a similar scenario to that described for paclitaxel, plant-derived leonurine limits its availability for use in research or in the clinical due to it occurring at low levels in plant tissues. While traditional separation and extraction methods do yield leonurine with higher purity (Deng et al., 2013; Cao et al., 2018), the amounts obtained are often low. Therefore, organic synthesis approaches are being employed to produce greater quantities of leonurine.

**FIGURE 7:**
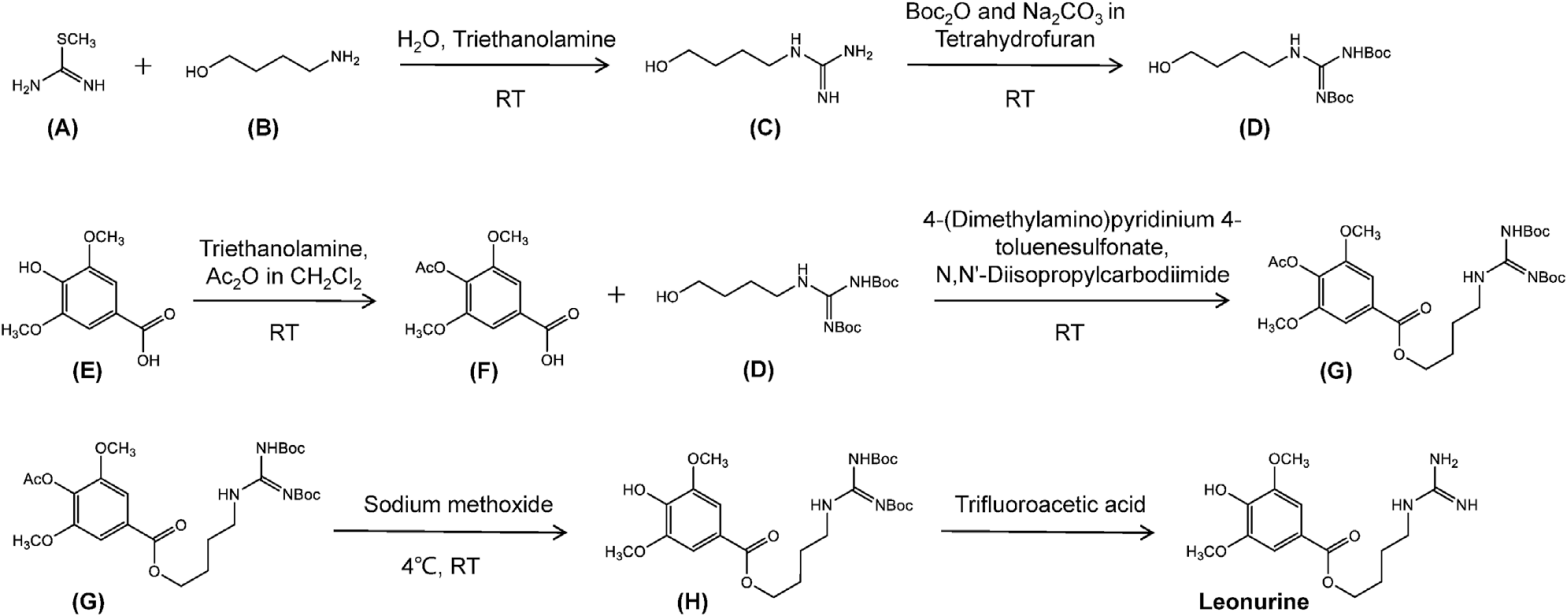
The chemical synthesis route of leonurine. RT, room temperature. Ac_2_O, acetic anhydride. Boc, tert-butoxycarbony group. **(A)** S-methyl-isothiourea. **(B)** 4-Amino-1-butanol. **(C)** N-(4-Hydroxybutyl). **(D)** Boc-protected N-(4-Hydroxybutyl)guanidine. **(E)** Caryophyllic acide. **(F)** 4-Acetoxy-3,5-dimethoxybenzoic acid. **(G)** Boc-protected 4-[(Aminoiminomethyl)amino]butyl 4-(acetyloxy)-3,5-dimethoxybenzoate. **(H)** Boc-protected leonurine.

The synthetic route used in the production of leonurine involves the preparation of the intermediary leucine urea, from succinic acid *via* the Gabriel reaction. This product is then reacted with S-methyl isothiourea sulfate to form leonurine (Cheng et al., 1979). This approach offers a simple method of production although the raw materials are rather expensive. This method has now been superseded using an optimized method developed in the laboratory of Zhu Yizhun at the University of Macau (Figure 7). The production of leonurine can now be achieved at low cost, and in high yield using S-methylisothiourea and 4-amino-1-butanol in a multistep synthesis. The compounds are protected using Boc anhydride to obtain an intermediate (D), and the phenolic hydroxyl group of caryophyllic acid acetic anhydride is used to obtain another key intermediate (F). Both (D) and (F) intermediates are further condensed to obtain a final intermediate (H), which is then deprotected under acidic conditions, to obtain leonurine (Cheng et al., 1979). This method produces large quantities of leonurine of high-purity and offers new sources of this compound for use in research or for clinical application.

### Pharmacological effects of leonurine

In mammalian models, leonurine is reported to promote blood circulation and overcome blood stasis (Miao et al., 2019), these properties are similar to the anticoagulant and anti-inflammatory effects of other traditional Chinese medicines (Deng et al., 1988). The anticoagulant effects have been reported to reduce the formation of thrombosis, reduce the risk of cardiovascular and cerebrovascular diseases such as atherosclerosis and myocardial infarction (Poredos et al., 2020; Alkarithi et al., 2021). This aroused the authors interest in leonurine and its potential use in cardiovascular and cerebrovascular diseases (Poredos et al., 2020; Alkarithi et al., 2021; Huang et al., 2021).

Cardiovascular disease is a complex multifactorial set of conditions with high mortality rate globally (Bozkurt et al., 2021). Long-term studies have shown that extracts of mother-wort have cardioprotective effect and can improve cardiovascular diseases, such as in models of atherosclerosis, myocardial infarction, and myocardial ischemia. In parallel, studies using purified leonurine are beginning to explore the efficacy of this compound in several clinical trials for the treatment of cardiovascular diseases. Indeed, atherosclerosis is the pathological basis of most serious cardiovascular diseases such as myocardial infarction and thrombosis, coupled with dyslipidemia; a key pathogenic risk factor linked to atherosclerosis. At present, the clinical treatment of atherosclerosis is largely based on the use of statins, but several side effects occur with this class of medication *viz.* impacts on muscle and severe liver function impairment (Suguro et al., 2018; Bozkurt et al., 2021). However, in the future other alternatives derived from natural products could be developed like leonurine. Leonurine has no apparent side-effects or adverse reactions when tested in various models and is effective at reducing atherosclerotic plaque formation, and attenuating atherosclerotic lesions by modulating inflammatory and oxidative stress pathways (Zhang et al., 2012). The pharmacological mechanism responsible for leonurine action to-ward inflammation and oxidative stress are complex are under investigation. Research by us and other groups, show that leonurine promotes cholesterol efflux by regulating the Pparγ/Lxrα signaling pathway, and attenuates the formation of atherosclerosis (Jiang et al., 2017). Moreover, leonurine not only reduced the occurrence of inflammatory response by inhibiting the activation of NF-κB (Liu et al., 2012), but also enhanced stress defenses in tissues including the activities of catalase (CAT), superoxide dismutase (SOD), glutathione peroxidase (GPx), and glutathione (GSH) levels to regulate oxidative stress (Zhang et al., 2012).

In addition to atherosclerosis, leonurine also improves myocardial infarction, an ischemic heart disease associated with cardiac damage and apoptosis. Leonurine protects cardiac function after myocardial infarction by increasing the viability of hypoxia-injured cardiomyocytes (Liu et al., 2009), by activating the PI3K/AKT/GSK3β signaling pathway (Xu et al., 2018), reducing the expression of pro-apoptotic genes including Bax and Bcl-2, and by inhibiting cell apoptosis (Liu et al., 2009). Similarly, leonurine also prevents cardiac fibrosis and cardiac fibroblast activation following myocardial infarction by regulating the Nox4-ROS pathway (Liu et al., 2013) and attenuate myocardial fibrosis after myocardial infarction by up-regulating miR-29a-3p. Combined these bioactive properties exerting cardio-protective effects in mammalian systems (Wang et al., 2021). More recently, a clinical phase I study has reported that leonurine alter the composition of intestinal microflora, and up-regulates the biosynthesis of adenosylcobalamin (AdoCbl). In turn, these actions promoted the conversion of homocysteine to methionine, reducing the levels of this proatherogenic sulfur amino acid (Liao et al., 2021).

Other research has shown leonurine to have significant therapeutic effects on diseases associated with the central nervous system including stroke, Alzheimer’s dis-ease, Parkinson’s disease, and depression syndrome (Huang et al., 2021). In the near future, clinical trials are being planned to assess leonurine in the treatment of central nervous system diseases. Stroke is one of the main types of cerebrovascular diseases seen in the clinic, that causes damage to brain tissues caused by cerebral ischemia and hypoxia (Kuriakose and Xiao, 2020). Research has shown that leonurine induces the antioxidant response by activating nuclear factor erythrocyte 2-related factor 2 (Nrf2), and upregulates the expression of vascular endothelial growth factor (VEGF) in neurons, astrocytes, and endothelial cells. Collectively, this prevents brain tissue ischemic injury (Xie et al., 2019). Moreover, leonurine was also shown to improve mitochondrial ultrastructure, to regulated mitochondrial function, and inhibited ATP synthesis, thereby exerting neuroprotective effects (Qi et al., 2010). Furthermore, researchers have shown that leonurine protects the integrity of the blood-brain barrier, and prevents stroke by regulating the HDAC4/NOX4/MMP-9 pathway (Zhang et al., 2017). In other neurological conditions like Alzheimer’s disease, Parkinson’s disease, and depression, leonurine likely acts by inhibiting neuro-inflammation. In other neurological conditions, leonurine promotes maturation of oligodendrocytes and enhancing the myelin sheaths in models of multiple sclerosis (Jin et al., 2019), inhibits the production of pro-inflammatory cytokines including interleukin one beta as well as interleukin 6, inhibits the nuclear factor kappa B signaling pathway (Jia et al., 2017), and promotes neurite outgrowth and neurotrophic activity by modulating the GR/SGK1 signaling pathway (Jia et al., 2017), thereby exerting an antidepressant effect.

Some evidence also points to other potential therapetuc effects in mammalian systems. Indeed, leonurine can inhibit PDZ-binding motif (TAZ) expression to regulate Treg/Th17 balance to alleviate rheumatoid arthritis (Du et al., 2020), it can inhibit PI3K/Akt/NF-κB signaling pathway to improve osteoarthritis (Yin and Lei, 2018), and improved renal fibrosis by inhibiting TGF-β and NF-κB signaling pathways (Cheng et al., 2015). Other studies show, leonurine can alleviate endometriosis by inhibiting the differentiation of regulatory T cells, providing a therapeutic approach for intractable diseases (Li et al., 2022). Taken together, this simple alkaloid appears to target multiple pathways linked to cytoprotection and inflammation in mammalian systems.

### Structure-activity relationship

**FIGURE 8:**
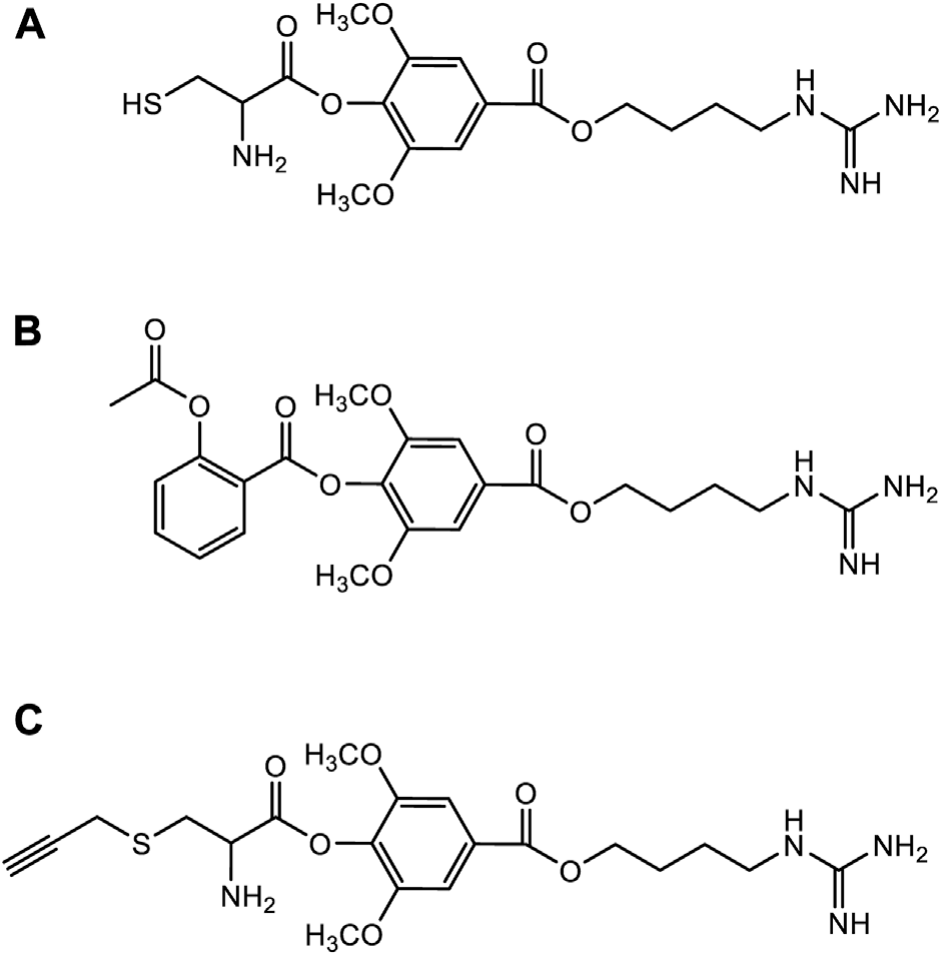
The structural modification of leonurine. **(A)** The chemical structures of leonurine-cysteine. **(B)** The chemical structures of leonurine-aspirin. **(C)** The chemical structures of leonurine-SPRC.

Although leonurine has great cardioprotective effects and has broad development prospects as a novel cardioprotective agent, it has certain difficulties in clinical application due to its unique chemical structure such as the guanidine group (Huang et al., 2021). Therefore, several medicinal chemists have been inspired by combination drug studies to study the structural modifications and structure-activity relationships (SARs) of leonurine. A study of SARs showed that the cardioprotective effect of leonurine was essential with butanolamine and guanidine group, and that the aromatic ring was tolerant to various substituents (Luo et al., 2020). Currently, the structural modification of leonurine mainly focuses on the combination with cysteine (Liu et al., 2011), aspirin (Gao et al., 2016), or S-propargyl cysteine (SPRC) (Luo et al., 2020) (Figure 8). Based on cysteine’s regulation of endogenous H_2_S through the cystathionine γ-lyase (CSE) pathway, Liu et al. designed a leonurine-cysteine analog conjugate. Leonurine-cysteine could modulate hydrogen sulfide production *in vivo*, enhance antioxidant activity, and have better anti-myocardial ischemia effects than leonurine (Liu et al., 2011). On the previous basis, Liu et al. further synthesized SPRC and combined leonurine and SPRC. The alkynyl group of SPRC is a strong electron-withdrawing group, and the carbon atom between the alkynyl group and the sulfur atom is more easily attacked by nucleophiles to generate cysteine, further releasing H2S. Leonurine-SPRC is also easily hydrolyze to release its bioactive substances, such as anti-oxidative stress and anti-apoptosis, and had effective cardioprotection against hypoxia-induced myocardial injury effect (Liu C. et al., 2010). Furthermore, Gao et al. designed a novel combination of leonurine-aspirin based on the antiplatelet activity of aspirin. It not only enhances antioxidant activity and protects cell membrane integrity, but also inhibits pro-inflammatory mediators for more efficient cardioprotection (Gao et al., 2016). So far, all of the novel compounds are more cardioprotective than either compound alone. Therefore, it is necessary to use leonurine as the parent nucleus to modify its structure to develop new novel drugs for cardioprotection.

## Conclusion and prospects

The current review summarizes some of the historical breakthroughs made using classical approaches to drugs discovery. In this instance, the natural products such as paclitaxel, artemisinin, aspirin, and camptothecin have been described. We also introduce, some of the work on the alkaloid, leonurine. Leonurine, is a novel natural product source, that is currently in development as a potential drug candidate. Work systematically summarized its development in recent times, including the plant origin, traditional therapeutic effects, chemical synthesis process, and rich pharmacological activities. Leonurine has attracted worldwide attention due to it having significant protective effects in the cardiovascular and neurological systems in mammals. Indeed, leonurine is now in the clinical trial stages of assessment. It is likely that this molecule, will become another example of how natural products can be exploited in modern day drug discovery programs. Hopefully, this series of stories will inspire new ideas for the development of natural products as drug candidates.
